# First detection of microplastics in *Xyrichtys novacula* (Linnaeus 1758) digestive tract from Eivissa Island (Western Mediterranean)

**DOI:** 10.1007/s11356-022-20298-8

**Published:** 2022-04-28

**Authors:** Amanda Cohen-Sánchez, Antònia Solomando, Samuel Pinya, Silvia Tejada, José María Valencia, Antonio Box, Antoni Sureda

**Affiliations:** 1 Research Group in Community Nutrition and Oxidative Stress (NUCOX), University of Balearic Islands, 07122 Palma de Mallorca, Balearic Islands Spain; 2 Interdisciplinary Ecology Group, Department of Biology, University of the Balearic Islands, 07122 Palma de Mallorca, Balearic Islands Spain; 3 Laboratory of Neurophysiology, University of the Balearic Islands, 07122 Palma de Mallorca, Spain; 4 CIBER Fisiopatología de la Obesidad y Nutrición (CIBEROBN), Instituto de Salud Carlos III (ISCIII), 28029 Madrid, Spain; 5 Health Research Institute of Balearic Islands (IdISBa), 07120 Palma de Mallorca, Spain; 6 LIMIA, Laboratori d’Investigacions Marines i Aqüicultura, 07157 Port d’Andratx, Spain; 7 Department of Agricultura, Ramaderia, Pesca, Caça i Cooperació Municipal, Consell Insular d’Eivissa, 07800 Eivissa, Spain

**Keywords:** *Xyrichtys novacula*, Marine debris, Plastic pollution, Microplastics, Mediterranean Sea

## Abstract

Plastic waste and its ubiquity in the oceans represent a growing problem for marine life worldwide. Microplastics (MPs) are ubiquitous in the sea and easily enter food webs. *Xyrichtys novacula* L. is one of the main target species of recreational fishing in the Balearic Islands, Spain. In the present study, the quantity of MPs in gastrointestinal tracts of *X. novacula* from two different areas (a marine protected area (MPA) and a non-protected area) of Eivissa Island (in the Balearic archipelago) has been assessed, as well as MPs evaluation within the sediment of both areas. The results showed that over 80% of sampled individuals had MPs in their gut with an average of 3.9 ± 4.3 plastic items/individual. Eighty percent of these plastics were fibres, while the rest were fragments. Although the sediment of the non-protected area had a significant higher presence of MPs, no significant differences in the number of MPs were observed in *X. novacula* from both areas. The µ-FT-IR analysis showed that the main polymers in the sediments were polycarbonate (PC) and polypropylene (PP), whereas in the digestive tract of fish PC, PP, polyethylene, polystyrene and polyester. In conclusion, practically all *X. novacula* specimens presented MPs in their digestive tract regardless if the capture zone was in a MPAs or not. These results highlight the ubiquity of MPs in coastal marine areas, and further studies might be necessary to evaluate further implications of MP presence in this species.

## Introduction

Plastic pollution is an increasing problem in the oceans today (Thushari and Senevirathna 2020). Since the beginning of plastic mass production in the 1950s, the amount of plastic manufactured has increased greatly, from 2.3 million tons in 1950 to 368 million tons in 2019 alone (PlasticsEurope 2020). An estimate of 8300 million metric tons (Mt) of virgin plastics have been produced as of 2017 (Geyer et al. 2017). This high continuous production of plastics has caused them to become ubiquitous pollutants in all marine habitats worldwide (Alimba and Faggio 2019; Horton and Barnes 2020). In 2010 alone, an estimate of 4 to 12 Mt of plastic waste generated on land entered the marine environment (Jambeck et al. 2015). Furthermore, since 2016, 150 Mt of plastic were estimated to already be circulating within the oceans (Schmaltz et al. 2020).

Marine plastic pollution involves many potential dangers related to ingestion (Gall and Thompson 2015), entanglement (Ryan 2018; Jepsen and de Bruyn 2019), facilitating the transport of invasive species and bacteria (De-la-Torre et al., 2021; Lewis et al., 2005), and even affecting the planktonic community (Fagiano et al. 2022). Moreover, plastics have a high resistance and little capacity to biodegrade, and over time they break down into small pieces of plastic, which will end up being microplastics (MPs) (Andrady 2011). These particles, generally defined as smaller than 5 mm (Thompson 2015), are found in aquatic systems all over the world (Barnes et al., 2009; Watt et al., 2021). Their source is both from originally small particles, such as fibres from clothing and components from beauty and health care products, and from the breaking down of macro plastics (Foley et al. 2018). Due to their small size, MPs become bioavailable via ingestion to marine organisms (Andrady 2011). As a matter of fact, MPs have become so common that they are found in almost all taxa (Foley et al. 2018), including zooplankton (Cole et al. 2013). Moreover, MP ingestion by fish has been widely assessed as a new matter of concern in the marine environments (Neves et al. 2015; Tanaka and Takada 2016; Lefebvre et al. 2019; Sun et al. 2019; Zhao et al. 2019; Zhu et al. 2019; Wu et al. 2020) and should be taken into account for their potential impacts on marine wildlife. In addition, plastics have additives in their composition such as plasticizers, flame-retardants, pigments and UV-stabilizers to give the plastic certain characteristics (flexibility, strength and colour) (Hermabessiere et al. 2017). Furthermore, because of their physical and chemical properties, plastics can accumulate chemical contaminants present in the seawater (Rochman et al. 2013). These additives and pollutants become available to the food chain and can enter cells, and, there, can interact and generate endocrine system disruptions (Alomar et al. 2017), as well as generate oxidative stress (Solomando et al. 2020).

*Xyrichtys novacula* (Linnaeus, 1758), or pearly razorfish, is a small wrasse which generally grows up to 20 cm (Schneider 1992). Its distribution is limited to warm latitudes of the Atlantic Ocean as well as the Mediterranean Sea (Castriota et al. 2005). It is highly benthic, associated to sandy bottoms (Cardinale et al. 1997; Katsanevakis 2005) where it dives headfirst into the sediment to protect itself and can remain buried for long periods of time (Alós et al. 2012). This species has been found to be territorial and with small home range in which it moves around randomly (Alós et al. 2012). *X. novacula* is a protogynous sequential hermaphrodite (Cardinale et al. 1997; Candi et al. 2004) with a clear sexual dimorphism (Battaglia et al. 2010). This species is gastronomically highly appreciated in the Balearic Islands, being one of the main targets of recreational fishing (Alós et al. 2012). In fact, the few times it is found in the market, its price is usually very high, or even the highest on the market (Beltrano et al. 2006). This led to overfishing, which forced the administration to impose a temporal ban, prohibiting the fishing of *X. novacula* from April to September (BOE-A-2000–7719 and later modifications BOE-A-2005–5315 and BOE-A-2011–9503).

This species has been found to feed on molluscs, crustaceans, echinoderms, other teleost fish (Beltrano et al., 2006; Cardinale et al., 1997; Luca Castriota et al., 2005) and colonial ascidians, as well as some species from the water column such as pelagic copepods, changing its diet and specialization with the resource abundance (Castriota et al. 2010). Therefore, it is known to feed mostly from the sediment where it buries and can also feed in the water column and has a very diverse diet (Castriota et al. 2005, 2010; Beltrano et al. 2006).

High-density plastics will naturally sink into the sediments, whilst buoyant plastics will stay in the water column. However, studies have proven that these low-density plastics suffer biofouling, allowing them to sink (Bellas et al., 2016). The continued use of plastics (Jambeck et al., 2015) and their tendency to sink with time, suggests the ocean sediment would have a large amount of accumulated MPs. Since *X. novacula* spends much of its life in and near the sediment and the fact that during its feeding the fish frequently ingests part of this sediment, it might be more exposed to MP ingestion than other fish with different life strategies. Furthermore, its wide array of prey makes it susceptible to capturing MPs from both benthic and pelagic environments. With all this, the aim of this work is to evaluate the presence of MPs in the digestive tract of *X. novacula* captured in two different areas of the island of Eivissa (Spain): a marine protected area (MPA) and a non-protected marine area, as well as its presence within the sediment of both areas.

## Material and methods

**Fig. 1:**
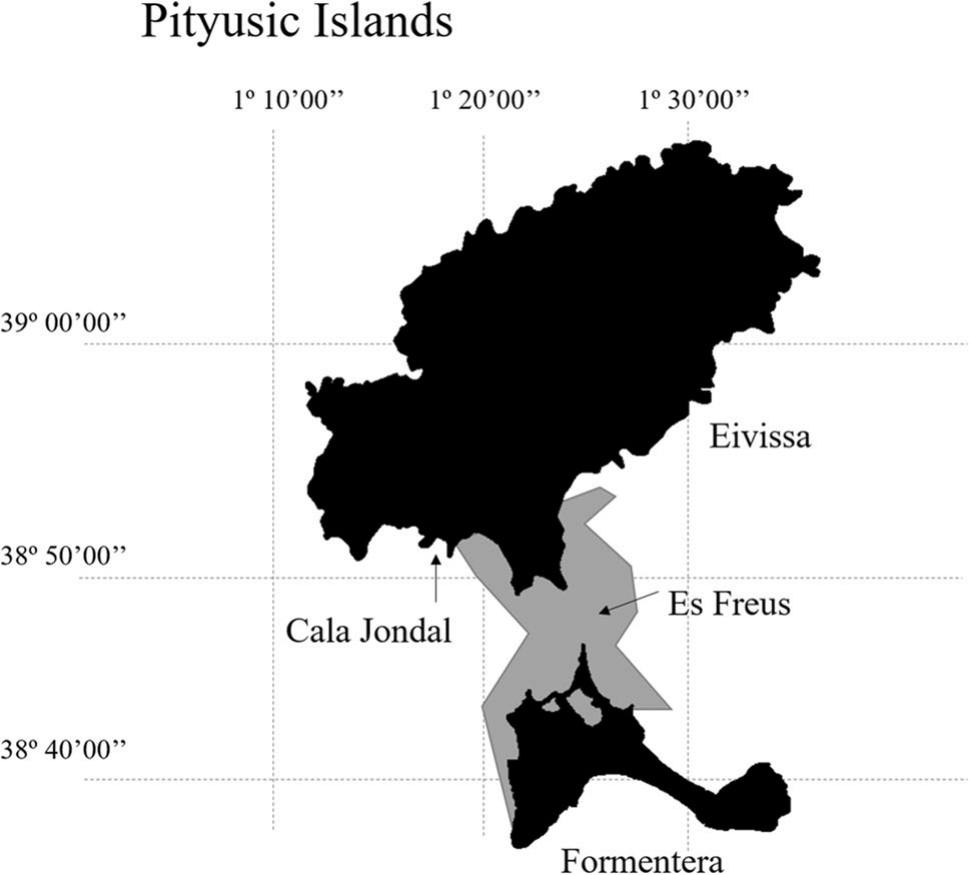
Localization of Eivissa Island (Balearic Islands, Spain, Western Mediterranean) with the two sampling zones: Es Freus, in a Marine Protected area and Cala Jondal, in a non-protected area). The area included in the Marine Protected Area is highlighted in grey colour

A total of 48 razorfish were fished around the island of Ibiza. Twenty-six were fished from the MPA area Es Freus and 22 from the non-protected area Cala Jondal by line fishing, using worms as bait (Fig. 1). Fish were captured during October 2020 to avoid the ban period, which protects the reproductive season of the fish. The fish were anesthetized with tricaine methanesulfonate (MS-222) (1 g/10 L water) to minimize stress. Individuals were measured and sexed and the whole gastrointestinal tract was dissected, weighed and frozen at − 20 °C until further analysis in the laboratory. The experimental procedure with fish has been carried out in accordance with the EU Directive 2010/63/EU for animal experiments and has been approved by the Ethics Committee for Animal Experimentation of the University of the Balearic Islands (Ref. 020/06/AEXP).

Gastrointestinal tracts were digested in Erlenmeyer flasks with 20 mL of 10% KOH solution per gram of digestive tissue (Dehaut et al., 2016; Thiele et al., 2019). After 24 h, the digested samples were filtered with a Büchner funnel, with filtering paper of 47-mm diameter and 10 µm of pore size (Albet LabScience, Barcelona, Spain) and the filters were introduced in a stove at 60 °C for 24 h. Control blanks were performed simultaneously under the same conditions as the samples. Then, the filters were taken out, and MPs were visually identified and photographed through a Leica EZ4 stereomicroscope with a HD camera (optical enhancement up to 11.5 ×). These MP particles were categorized via visual observation by colour, size and type (fibre or fragment). During the handling process, measures to avoid microplastic contamination were adopted, such as minimizing the contact of the sample with the air to avoid airborne fibres working under a laminar flow hood, as well as rinsing and cleaning material and surfaces using filtered ethanol 70% and MilliQ water. The samples were covered with aluminium foil during digestion, stirring, decantation and filtration steps. In addition, lab coats made of 100% cotton were worn throughout the experiments (Woodall et al. 2015).

For the sediment analysis, sand samples were taken from both study areas by experimented divers. An auger was employed to collect the sediment, inserting it into the sediment surface at a 0–45° angle from vertical to minimize the loss of surface sediment and water entering. Because MPs were being sampled, there was no concern in mixing the sediment, which was then stored in clean glass and pre-labelled containers (Tuit and Wait 2020). In the laboratory, 250 g of sediment was mixed with a previously filtered saline solution (1 L H_2_O + 120 g NaCl) to generate a dense solution that allowed the MPs to float (Claessens et al. 2011). The mix was stirred for 2 min and the sand particles were allowed to settle for 1 h. Then, the saline solution with floating MPs was filtered, dried and MPs were visually identified using the same methodology as for the digestive tracts.

The granulometry and organic matter present in the sediment were also determined. For granulometry, 200-g sediment sample from both sites were dried in a heater at 100 °C and were later sieved through sieves of sizes 2 mm, 1 mm, 500 µm, 250 µm, 125 µm, 63 µm and < 63 µm. For organic matter analysis, 100 g of sediment was weighed and placed into pre-weighed cups made from heavy-duty aluminium foil. The dried samples are cooled in a desiccator for 15–20 min and weighed again, before being muffled at 350 °C overnight to remove organic matter. The combusted sample was weighed and the difference in weight accounted for the organic matter.

Chemical identification of polymer types was run on a minimum subset of 10% of the identified items (54 plastic items). These items were analysed by attenuated total reflection micro-Fourier-transform infrared spectroscopy (µ-ATR-FTIR) (Bruker, OPTICS, Germany) to determine polymer types. Due to the small size of the MPs, analysis was performed by the Hyperion ATR microscope. The MPs were identified visually within the filter and analysed by the spectrometer. Wave number range between 400 and 4000 cm^−1^ and 250 scans were performed per item. Each spectrum was subjected to a baseline correction and compared with commercial and custom-made spectral databases. A minimum of 700 hit quality index was necessary to accept a confirmed polymer (Bergmann et al., 2017). The analysis was carried out with the support of the Scientific/Technical Services of the University of Balearic Islands. In addition, those items in which their plastic origin was doubted were also confirmed with µ-ATR-FTIR.

Data was treated with Excel and statistical analysis was carried out with R. Normality was tested with a Lillie test and homogeneity of variance with the Levene test. Due to lack of normality, the comparisons were done with Kruskal Wallis, and correlations were calculated with Pearson’s test. Data are expressed as mean ± SEM and median (interquartile range) for continuous variables and as counts (percentages) for categorical variables. Statistical significance was set at *p* < 0.05.

## Results

A total of 48 fish were caught (22 in Cala Jondal and 26 in Es Freus), with an average size of 14.73 ± 2.58 cm and weight of 38.92 ± 25.06 g. Fish were caught at a depth between 15 and 20 m, 19 °C and 36 psu.

**Fig. 2:**
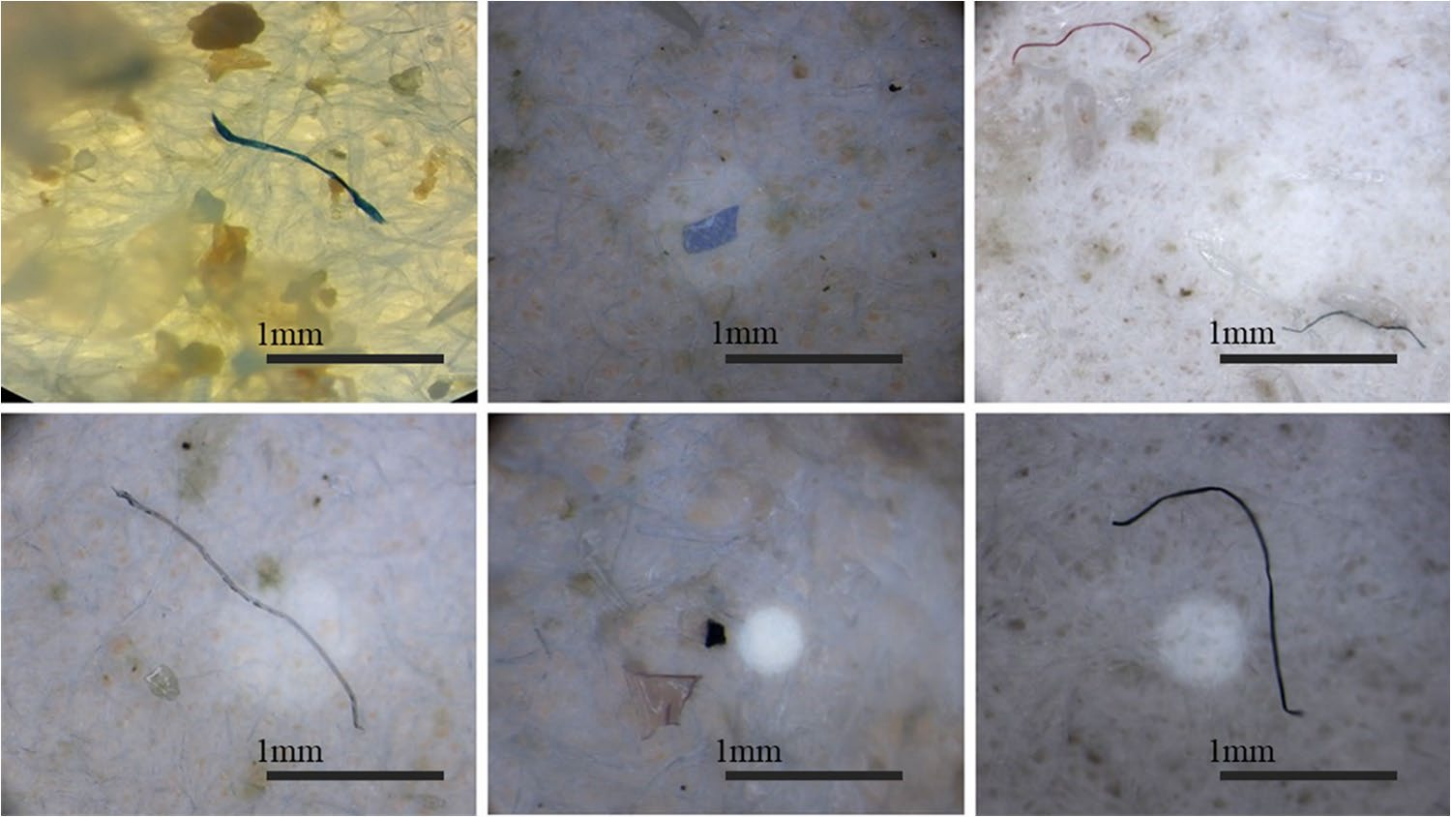
Representative images of the microplastics found in the gastrointestinal tract *Xyrichtys novacula* sampled in Eivissa Island (Spain) taken by the Leica EZ4 stereomicroscope with a HD camera. Scale bar represents 1 mm

MPs were found in the gastrointestinal tract of 46 of the sampled fish (91%), whilst only the remaining 2 (9%) were MP-free. Figure 2 shows representative images of the MPs observed in the gastrointestinal tracts of the fish. On average, the fish had 3.64 ± 0.38 items/individual in their gastrointestinal tract.

**Fig. 3:**
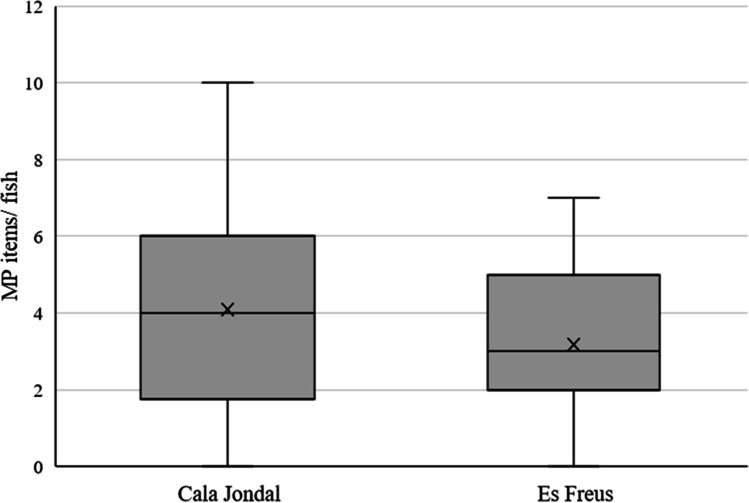
Microplastic items per individual of *Xyrichtys novacula* collected in Cala Jondal (non-MPA) (*n* = 22) and in Es Freus (MPA) (*n* = 26) (Eivissa, Spain). Statistical analysis was carried out with Kruskal Wallis test. No significant differences between areas were evidenced

When analysing the differences in the content of MPs between the fish caught in the two study areas, no significant differences were observed (*p* > 0.05). The average MPs content for the non-protected area an average of 4.06 ± 0.62 items/individual and for the protected area is 3.18 ± 0.43 items/individual (Fig. 3). Among the fish, 43% of them had 2 or less MPs/individual, 35% had between 3 and 5 MPs/individual, and 10% had over 5 MPs items/individual. No noteworthy correlations were found for the number of MPs with respect to fish size (*r* =  − 0.27) or weight (*r* =  − 0.18).

**Fig. 4:**
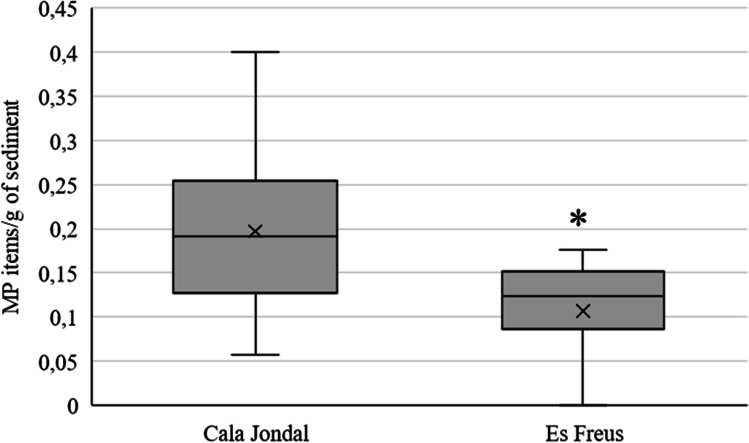
Microplastic items per gram of sediment collected in Cala Jondal (non-MPA) and in Es Freus (MPA) (Eivissa, Spain). Statistical analysis was carried out with Kruskal Wallis test. Cala Jondal has a significantly higher amount of microplastics than Es Freus, * *p* < 0.05

The presence of MPs in the sediment of the two study areas is shown in Fig. 4. Significant differences in the presence of MPs were found between both areas (*p* < 0.01), with an average of 0.19 ± 0.02 items/g sediment in Cala Jondal, and an average of 0.11 ± 0.01 items/g for Es Freus.

**Fig. 5:**
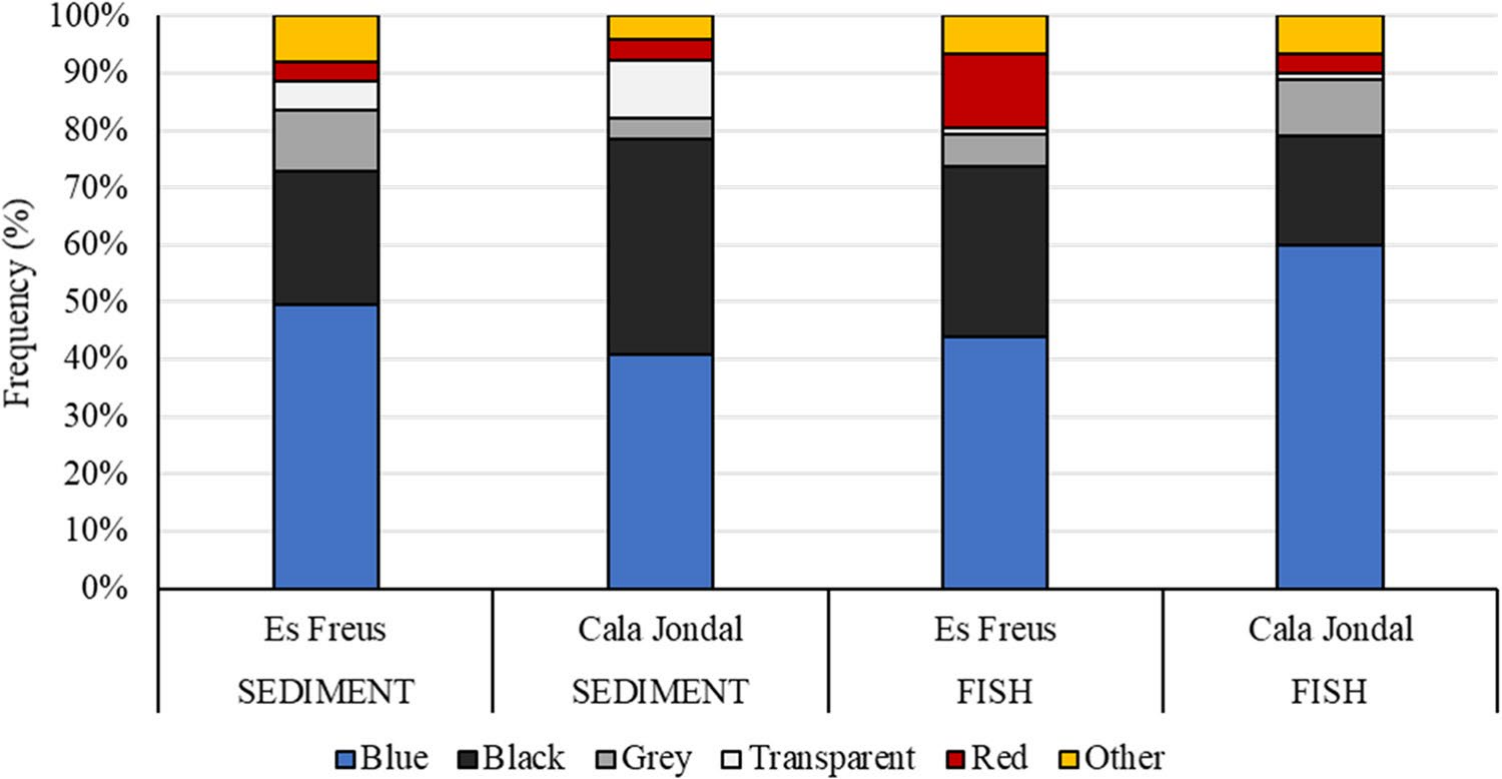
Proportion of plastic colours found in *Xyrichtys novacula* gastrointestinal tracts (FISH) and sand samples (SEDIMENT) in both the MPA (Es Freus) and the non-MPA (Cala Jondal) sampled in Eivissa Island (Spain)

**Fig. 6:**
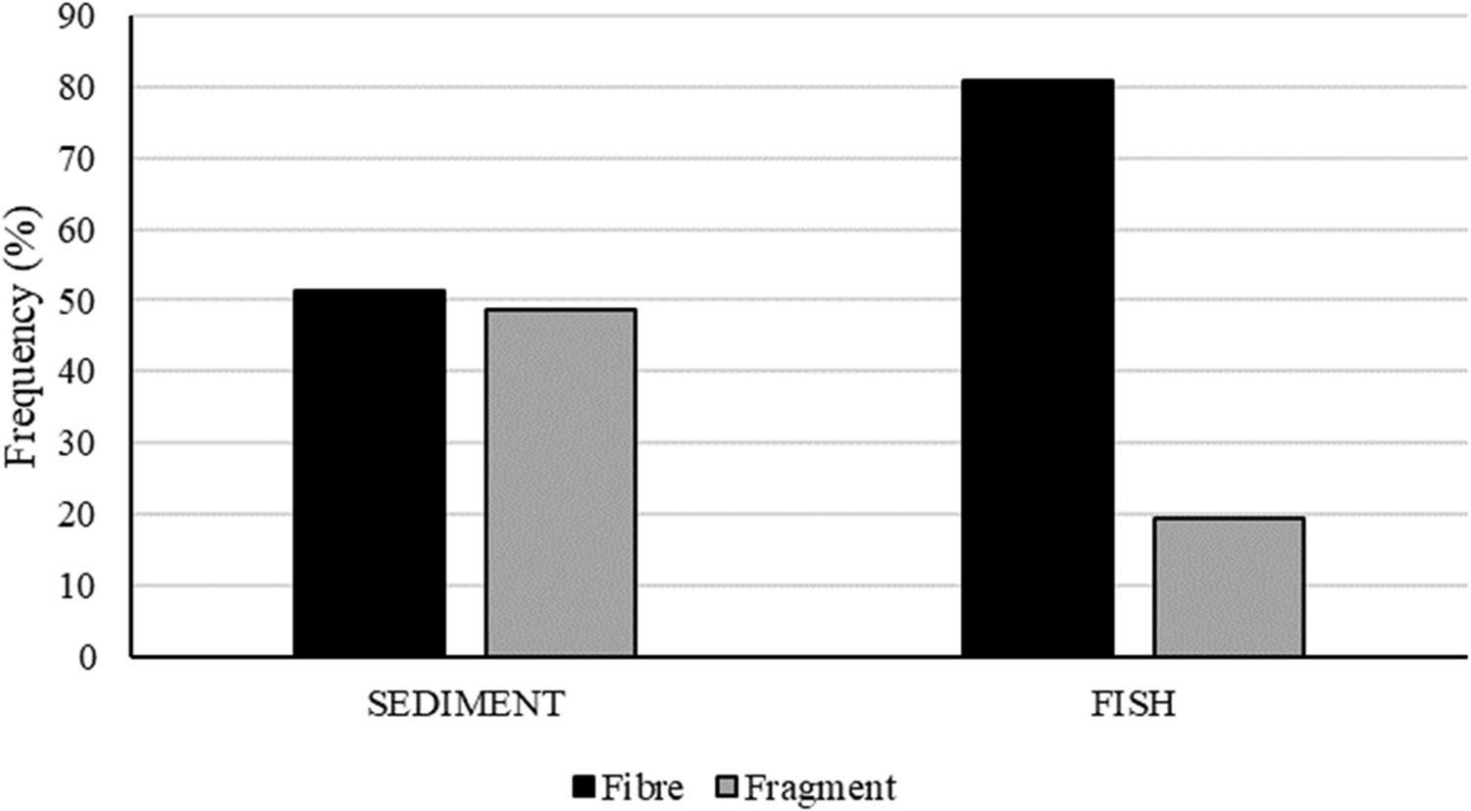
Proportion of plastic type (fibre/fragment) found in *Xyrichtys novacula* gastrointestinal tracts (FISH) and sand samples (SEDIMENT) in Eivissa Island (Spain)

Regarding the characteristics of the MPs found, for fish, 80% of the particles identified were fibres, while the rest were small fragments. For sediment, 51% were fibres and 49% fragments (Fig. 5). As for colours, the most common MP colour in all samples from fish and sediment was blue (50–60%), followed by black (40–20%) (Fig. 6).

**Fig. 7:**
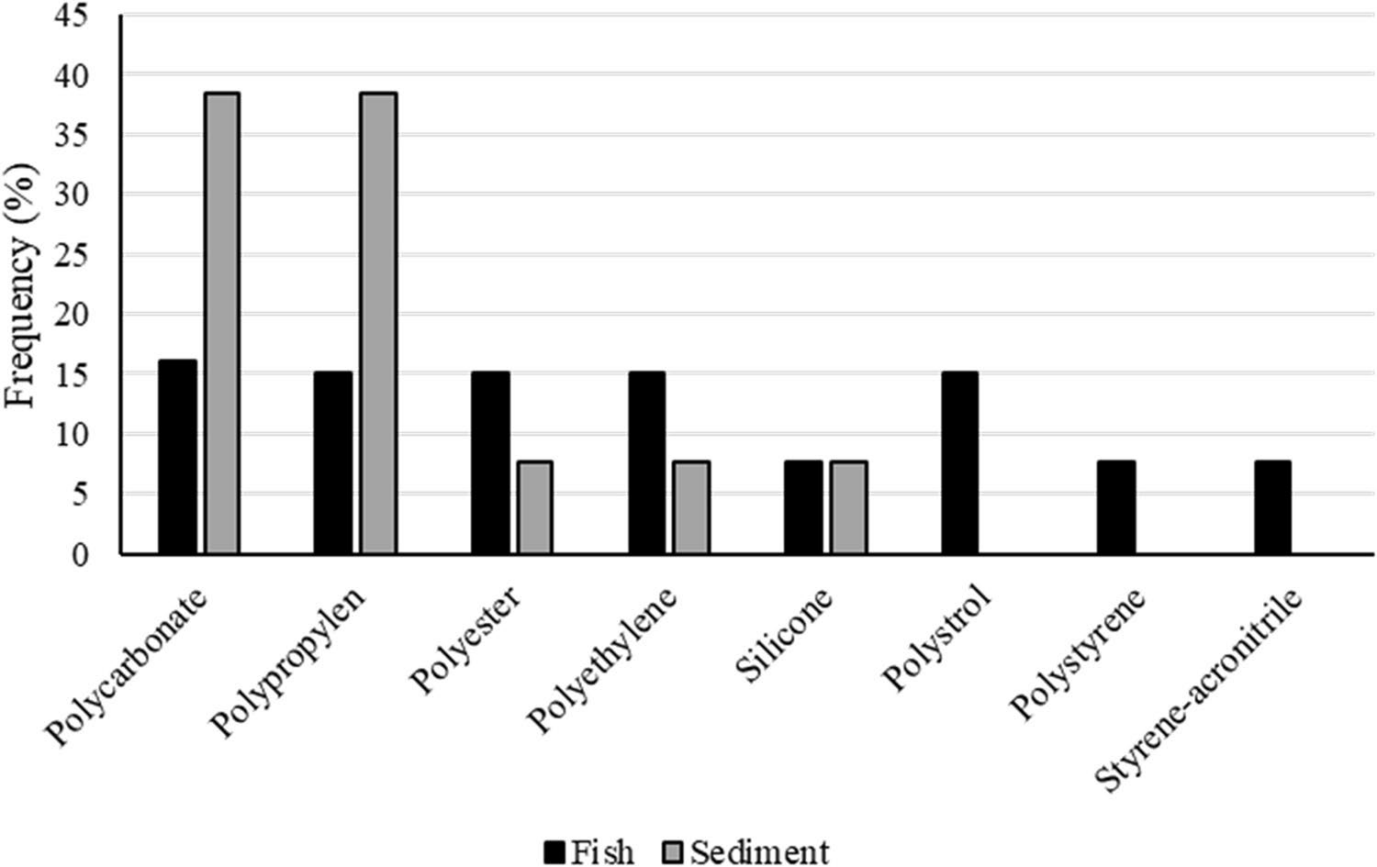
Proportion of plastic polymer type found in *Xyrichtys novacula* gastrointestinal tracts (FISH) and sand samples (SEDIMENT) in Eivissa Island (Spain)

A total of 54 items were analysed with µ-ATR-FTIR for chemical polymer identification. For fish samples, the most common polymers were polycarbonate (PC, 16%), polyester (PET, 15%), polyethylene (PE, 15%), polypropylene (PP, 15%) and polystyrene (PS, 15%), adding up to 76%. For sediment samples, the most frequent polymers were PC (38%) and PP (38%), followed by PE (19%) (Fig. 7).

In the characterization of the sediments, the granulometry showed that in both zones the particle size is mostly between 125 and 250 µm, with more than 90% included in this range, which would indicate that they are sandy bottoms. The organic matter found was very low (less than 3%) and, therefore, considered negligible. No differences were found between organic matter quantity within both the MPA and non-MPA areas (*p* > 0.05).

## Discussion

*Xyrichtys novacula* is a highly appreciated fish in the traditional gastronomy of the Balearic Islands; thus, analysing possible factors that may affect their populations and healthy status is of relevance. Among the threats, plastics have recently gained relevance due to their ubiquity and capacity to cause physical and mechanical damage, inflammation, obstruction of the gastrointestinal tract, alter reproduction and change the behaviour in exposed organisms (Jovanović 2017; Foley et al. 2018; Fossi et al. 2018). The results obtained provide newfound evidence of the presence of MPs in gastrointestinal tract of *X. novacula,* as well as the presence of MPs in practically all the specimens of *X. novacula* from Eivissa Island (Spain), regardless of whether they have been captured in a MPA or in a non-protected area.

The ubiquity of MPs greatly favours its ingestion by animals, whether directly mistaken for a prey, by accident (associated with or attached to prey), or via absorption through gills (Foley et al. 2018). The presence of MPs in over 90% of the gastrointestinal tracts of *X. novacula* is higher than most of the results reported in other studies. In this sense, Neves et al. (2015) reported that 19.8% of commercial fish from the Portuguese coast presented MPs (*N* = 263, 26 different species) with *Scomber japonicus* (Houttuyn 1782) reporting highest mean of ingested microplastics (31%). In the study of Alomar et al. (2017), the authors described MPs in 27.3% of *Mullus surmuletus* (Linnaeus 1758) (*N* = 417) captured off the coast of Mallorca island, Spain. In addition, results by Güven et al. (2017) evidenced that 58% of the sampled individuals representing 28 species from Turkish waters had MPs (*N* = 1337) *Siganus luridus* (Rüppell 1829) and *S. japonicus* being the species with more MPs (67 and 57%, respectively). The present results are more similar to those of Zhu et al. (2019), who found that 100% of the sampled fish presented microplastics (*N* = 35, 11 species) in deep-sea fish from the South China Sea. Also, Tanaka and Takada (2016) found MPs in 77% of *Engraulis japonicus* (Temminck and Schlegel 1846) (*N* = 64) sampled in Tokyo Bay (Japan), whereas Nadal et al. (2016) found that 68% of *Boops boops* (Linnaeus 1758) sampled in Mallorca coastal waters had MPs (*N* = 337). Species such as *B. boops*, *Engraulis encrasicolus*, *Sardina pilchardus*, *Scomber colias*, *Scomber scombrus* and *Trachurus trachurus* from the Portuguese coast also present a high MP occurrence, the lowest being 58% for *S. pilchardus* and the highest 100% for *S. scombrus* and *T. trachurus* (Lopes et al., 2020). This high occurrence of MPs in the gastrointestinal tract of *X. novacula*, together with its benthic lifestyle and frequently ingest sediment when feeding, makes this species an interesting study subject for MP presence, ingestion and effects.

As for quantity of MPs found per individual, the number (3.6 ± 0.38 plastic items/individual) was also higher in this work than the results from other studies. For example, Lusher et al. (2013) found an average of 1.9 plastic pieces in pelagic and demersal fish from the English Channel, whereas Tanaka and Takada (2016) found 2.3 pieces on average and up to 15 pieces per individual of *E. japonicus* from Tokyo Bay. On the contrary, several studies reported similar mean values of MPs to those obtained in *X. novacula*. For instance, a mean of 3.8 MPs/individual was reported in deep-sea fish from the South China Sea (Zhu et al. 2019) or 3.8 items in *B. boops* captured in Mallorca coastal waters (Nadal et al. 2016).

The differences in the number of MPs ingested seem to depend, in part, on the feeding strategy of the fish. The strategies used by non-selective fishes that feed on small prey may expose them to high MPs ingestion levels (Mercogliano et al. 2020). Thus, for similar sized fish, *Engraulis encrasicolus* (Linnaeus, 1758) is a selective feeder, while *Sardina pilchardus* (Walbaum 1792) is a filter feeder and, consequently, unable to select the ingested particles, and therefore presents a higher content in MPs (Renzi et al. 2019). Furthermore, opportunistic feeder fish such as *Gadus morhua* (Linnaeus 1758), which may feed on a wide variety of prey, can be also more exposed to the ingestion of anthropogenic particles (Hansen et al. 2016). In accordance, *X. novacula* is a fish that feeds on very diverse prey and this trophic plasticity could contribute to the relevant presence of MPs in its gastrointestinal tract. Moreover, the fact that almost 50% of the ingested MPs found were blue, and 24% were black, suggests that these colours could be ingested by confusion due to the similarity to common prey (Lopes et al. 2020). In addition, these results can also be associated with a greater presence of black and blue plastics in the ocean, which could come from the land, from the fiber of clothing, packaging and plastic containers. Similar results were obtained in *M. surmuletus* from Balearic Islands (Alomar et al. 2017) in a wide diversity of fish species from Turkish coastal waters (Güven et al. 2017), in wild fish from the North East Atlantic Ocean (Barboza et al., 2020), and planktivorous fish off the coast of Portugal (Lopes et al., 2020). In the present work, 81% of MPs found in pearly razorfish were fibres. These results match those of Nadal et al. (2016) in their study with *B. boops* also carried out in the Balearic Islands. In fact, according to López-Martínez et al. (2021) that analysed data from around the world, fibres were the predominant type of MPs in fish with more than 65% presence. Lopes et al., (2020) also found a predominance of fibres. This majority of fibres can derive from textiles, hygiene and cosmetic products, and the fishing industry that reach the sea mainly from the washing discharge around the archipelago (Andrady 2011). Specifically, in 2016, 23 water treatment plants were operating around Eivissa, 12 of them discharging directly into the sea. This discharge cannot always be subject to tertiary treatment (only about 65%), and this amount decreases with the tourism pressure, leading to massive discharges directly into the sea (Del Valle et al. 2017).

When characterizing the sediments of the two selected areas, it is observed that both are made up of sandy bottoms and with little presence of organic matter. Regarding MP analysis, the results obtained show that in the MPA, characterized by a lower anthropogenic impact, the number of MPs is lower than in the non-protected area. Es Freus is a MPA with various protection levels, but with human impact, such as boat traffic. However, according to data provided by the Balearic government, several sewage disposal points were located near the non-protected area. Consequently, this area would therefore be more susceptible than the MPA to MP accumulating in the sediment.

*X. novacula* is a territorial fish with a small home range (Alós et al., 2012) and therefore the MP presence in their gastrointestinal tract is likely to be indicative of their environment, as they will not move great distances to feed. However, despite the varied diet of *X. novacula* and its extensive predatory strategy that includes many benthic species, the MPs in the sediment do not seem to affect MP content in the fish. The absence of differences between the two capture areas may be due to the ubiquity of MP, and the lack of physical barriers to impede their spreading. It could also be due to a short residence time of the MPs in the digestive tract of the fish. These results are supported by the absence of any correlation between the size / weight of the fish and the content of MPs. Similarly, other studies including many different types of fish species, concluded that fish length and mass are not factors which influence MP presence (Neves et al. 2015; Güven et al. 2017). In a study carried out with *Seriolella violacea* (Guichenot 1848)*,* Ory et al. (2018) in a laboratory under controlled conditions observed that MPs remained an average of 7 days in the gastrointestinal tract before being egested. Thus, the presence of MPs in fish would be indicative of the intake of these elements in a relatively recent time, rather than being indicative of bioaccumulation processes. However, some studies have proven bioaccumulation of MP and their additives in different fish tissues (W. Liu et al., 2020; Xu et al., 2020). These MPs and the high amount of additives that these plastics have can affect different species, as they can be absorbed at the intestinal level (Zhu et al. 2019).

Microplastic presence in the marine environment can occur through various sources and pathways, including plastic manufacturing, water and treatment plants and industrial waste (Issac & Kandasubramanian, 2021). The most common polymers present in sediment are PP and PC. PC is a durable and malleable polymer widely used across a variety of industries, with versatile applications, but is also susceptible to photooxidation and mechanical abrasion which could lead to fragmentation and MP accumulation in the ocean (Shi et al., 2021). This polymer has been found in sediment analysis (Zhang et al., 2021) and also sewage sludge, a large proportion of which is estimated to reach the ocean (Zhang et al., 2019). PP is a rigid and crystalline thermoplastic that is used in a wide variety of domestic and industrial applications, from packaging to parts for machinery and equipment, and even fibres and textiles. Although PP is a low-density polymer (Suaria et al., 2016), biofouling can favour its deposition (Morét-Ferguson et al., 2010), and it can be found in beach sediments (Tiwari et al., 2019), in sand from a protected natural park (Scopetani et al., 2021), and even in the Mariana Trench (Peng et al., 2018). As for polymers found in fish gastrointestinal tracts, there is much more variety, although the polymers identified through µ-FT-IR spectrometry are some of the most common in plastic production and daily plastic use (Geyer et al., 2017; Liu et al., 2021). PE, PP and PET are the three most manufactured and used plastics globally (Leng et al., 2018), therefore it is no surprise to find them in fish digestive tracts. All of these polymers, as well as others, have been reported within fish (Tanaka & Takada, 2016). PE and PET are the most common polymer found in wild fish in the North East Atlantic Ocean (Barboza et al., 2020), PE, PET and PP are also found to be abundant in a meta-analysis of different studies (Miller et al., 2020), and in benthic and epibenthic species in Norway, being PP and PE the most frequent polymers (Bour et al., 2018). Nevertheless, further studies including a more exhaustive analysis of MPs in water column, sediment and fish digestive tracts in different points around the island could be interesting to better understand the source of the ingested MPs by *X. novacula*.

## Conclusions

Results from this study provide newfound evidence of the ingestion of MPs by *X. novacula*. Most of the sampled fish contained MPs and the number of MP per fish was high in relation to other studies, thus making it an interesting species to study in relation to plastic effects and intake. This quantity, however, does not seem to depend on the area of capture, size or weight of fish. Although the non-protected area presented a higher number of MPs in the sediment, this fact was not reflected in a higher content in *X. novacula* caught in this area, suggesting that the residence time of the MPs in the digestive tract is relatively short. As for plastic polymers, those found in *X. novacula* are some of the most manufactured plastics globally, such as PC, PE and PET. More studies regarding the relation between MP content in the digestive tracts and in the marine environment are needed to further understand the dynamics of MP ingested by fish.

## Data Availability

The datasets generated during and/or analysed during the current study are available from the corresponding author on reasonable request.
